# Validation of the comprehensive feeding practice questionnaire among school aged children in Jordan: a factor analysis study

**DOI:** 10.1186/s12966-017-0478-y

**Published:** 2017-02-20

**Authors:** Walid A. Al-Qerem, Jonathan Ling, Abdul Qader AlBawab

**Affiliations:** 1 Al-Zaytoonah University of Jordan, P.O. Box 130, Amman, 11733 Jordan; 2 Department of Pharmacy, Health and Wellbeing, Faculty of Applied Sciences, University of Sunderland, Chester Rd, Sunderland, SR1 3SD UK

**Keywords:** Childhood obesity, Jordan, Feeding practices

## Abstract

**Background:**

Obesity has become a significant worldwide contributor to morbidity with an alarming increase in the incidence of childhood obesity. Few studies have evaluated parental feeding practices and their impact on child obesity in the Middle East. The Comprehensive Feeding Practice questionnaire (CFPQ; Musher-Eizenman & Holub, 2007) has been validated in different age groups and in different countries, however no previous studies have validated the questionnaire in the Middle East.

**Method:**

In this study, 970 children aged 6–12 completed the Arabic translated version of the CFPQ. The height and weight of the children were also measured. Confirmatory factor and exploratory factor analysis were used to evaluate different factor models. An ordinal logistic regression was conducted to evaluate the association between maternal feeding practices and child weight status.

**Results:**

Confirmatory analysis of the CFPQ determined that the original 12 factor structure of the questionnaire was not suitable for this sample. The analysis suggested that the most suitable structure was an 11 factors model (CMIN/DF = 2.18, GFI = 0.92, CFI = 0.93, TLI = 0.92 and RMSEA = 0.03) that included *Modelling, Monitoring, Child control, Food as a reward, Emotional regulation, Involvement, Restriction for health, Restriction for weight control, Environment, Teach and encourage* and *Pressure*. Of the children tested, 12.6% were obese and 25.1% were overweight. The ordinal regression showed *Restriction to health and weight, Emotional regulation* and maternal BMI were negatively associated with healthy weight status, while *Modelling, Monitoring, Child Control, Environment, Involvement,* and *Teach and encourage* were positively associated with healthy weight status.

**Conclusion:**

The Arabic translated version of the CFPQ was validated among the study sample, and the best fit for the model was found to utilize 11 factors. This study indicated that child weight status was associated with maternal feeding practices.

**Electronic supplementary material:**

The online version of this article (doi:10.1186/s12966-017-0478-y) contains supplementary material, which is available to authorized users.

## Background

Obesity has become a major health issue worldwide with obesity increasing in all age groups. Jordan and the Middle East in general had high reported obesity prevalence rates, for example according to World Obesity [1], 21.1% of boys aged 15–18 in Jordan are overweight and 10.2% are obese. In the neighbouring country of Kuwait 60.4% are overweight or obese. Other work found that among Jordanian children aged 6–12, 19.4% were overweight (18.8% of boys and 19.9% of girls) and 5.6% were obese (5.6% of boys and 5.5% of girls) [2]. These are high rates and emphasise the need for monitoring and interventions to reduce overweight and obesity in this age group.

Although studies have established the relationship between parental feeding practices and children’s weight [3], the impact of several feeding practices on child weight are still inconclusive [4], for example contradictory findings were reported when evaluating the association between monitoring children’s food intake and their weight status [5, 6]. Several questionnaires have been proposed to evaluate this relationship, with one of most frequently used being the Child Feeding Questionnaire [7] which includes seven factors. Four factors evaluate parental perceptions and concerns that may lead parents to control their child feeding practices (i.e., perceived parent weight, perceived child weight, parental concern about child weight, and parental responsibility). The other three factors evaluate parental control attitudes and practices in child feeding including the use of restriction, pressuring children to eat more, and monitoring.

A more comprehensive questionnaire, the Comprehensive Feeding Practice Questionnaire (CFPQ) [8] was developed to cover other domains that may affect parental feeding practices. The CFPQ includes 12 factors and is composed of 49 items that cover feeding guidance, restriction and pressuring, using food to regulate behaviour and providing an appropriate environment (the availability of healthy food). Such a questionnaire can explore extensive information about childhood overweight and obesity, and factors that potentially influence children’s habits that may contribute to the problem.

The first aim of this study was to develop an Arabic version of the CFPQ and to examine its validity in a large sample of schoolchildren aged 6–12 years old in Jordan. The validated questionnaire can then be applied in the different territories of the Arabic speaking language area throughout the Middle East and North Africa; allowing researchers and health authorities to examine childhood overweight and obesity and develop an understanding of potential solutions, as currently there is no work in this region. Furthermore, the validated Arabic version of the CFPQ can be utilized in future epidemiological studies, which are lacking in the Middle East and North Africa geographical region. The second aim of this study to evaluate the association between different maternal feeding practices and children’s weight in Jordan, and as such this is one of the first studies to focus on this issue conducted within the Middle East.

## Methods

### Participants

The children recruited for this study came from five primary schools in Madaba Governorate in Jordan that is located south of the capital Amman with a population of 189,192 [9]. Madaba is representative of wider Jordan because of it is proximity to the capital city, the diversity of its inhabitants, that includes Christians and Muslims and it also includes a Palestinian refugee camp. Madaba has both rural and urban areas: the northern region of the governorate is agricultural, that mainly cultivates fruit and olives [10], while inhabitants of Madaba city have an urban lifestyle.

Access to schools was granted by the Ministry of Education. Children and their parents/guardians were fully informed and the parents signed a consent form. The questionnaire was completed by the children’s’ mothers. The translated questionnaire (see Additional file 1) was circulated with the consent form to 1,350 children from 5 schools located in different parts of Madaba governorate. Different approaches were made to evaluate the appropriate sample size for conducting confirmatory factor analysis and exploratory factor analysis (EFA); CFA is a statistical technique used to verify the factor structure of a set of observed variables. CFA allows the researcher to test the hypothesis that a relationship between observed variables and their underlying latent constructs exists [11]. EFA is a statistical technique used to explore the possible underlying factor structure of a set of observed variables without imposing a preconceived structure on the outcome [12]. When determining the appropriate sample size some focus on the total number while others focused on the subject to item ratio. The studies that focused on the sample size suggested numbers ranging from 50 [13] to 1000 to achieve an adequate sample size [14]. One frequently used guide is having a participant to item ratio of 20:1 [15]. Therefore, the sample size selected for this study was almost 1000 subjects to achieve the conditions suggested by the two approaches.

### Materials

The CFPQ [8] includes 49 questions divided into 12 domains. These are child control his eating behaviour *(Child control)*, usage of food by parents to regulate the child’s emotional states (*Emotion regulation)*, parents promoting well-balanced food intake *(Encourage balance and variety)*, parents making healthy foods available in the home *(Environment)*, parents using food as a reward for child’s behaviour *(Food as reward)*, parents encouraging child’s involvement in meal planning and preparation *(Involvement)*, parents actively demonstrating healthy eating for the child *(Modelling)*, parents keeping track of child’s intake of less healthy foods *(Monitoring)*, parents pressuring the child to consume more food at meals *(Pressure)*, parents controlling the child’s food intake with the purpose of limiting less healthy foods and sweets *(Restriction for health)*, parents controlling the child’s food intake with the purpose of decreasing or maintaining the child’s weight *(Restriction for weight control)*, and parents using explicit didactic techniques to encourage the consumption of healthy foods *(Teaching about nutrition)*.

The 12 factors were constructed from 49 items with two response formats. The first 13 questions had a 5-point response scale “never, rarely, sometimes, mostly, and always”. The remaining questions had a 5-point scale, “disagree, slightly disagree, neutral, slightly agree, and agree”. Questions number 16, 37 and 42 were reverse coded. The CFPQ was translated into Arabic and back translated to English. The back translated version was compared with the original English questionnaire by a native English speaker and further changes were made where necessary (see Table 1 and Additional file 1 and Additional file 2).

**Table 1:** CFPQ structure

Factor	Item^a^
Monitoring	1, 2, 3, 4
Child Control	5,6,10,11,12
Emotional regulation	7, 8, 9
Environment	14, 16^b^, 22, 37^b^
Involvement	15, 20, 32
Pressure	17, 30, 39, 49
Restriction to weight	18, 27, 29, 33, 34, 35, 41, 45
Food as a reward	23, 36. 19
Restriction to health	21, 28, 40, 43
Modelling	44, 46, 47, 48
Teaching about nutrition	25, 31, 42^b^
Encourage balance and variety	13, 24, 26, 38

^a^The first 13 questions had a 5-point response scale “never, rarely, sometimes, mostly, and always”. The remaining questions had a 5-point scale, “disagree, slightly disagree, neutral, slightly agree, and agree”

^b^Reverse coded

Additional questions were added at the beginning of the original questionnaire. These questions consisted of child’s gender, child’s age, mother’s education level and self-reported mother’s height and weight. Maternal BMI was calculated and maternal weight status was determined according to the WHO classification [16]; obesity was defined by BMI greater than 30 and overweight was defined by BMI 25.0–30.0 and underweight was defined by BMI less than 18.5. Children’s BMI were calculated. Child weight status was determined using the International Obesity Task Force (IOTF) standard points which sets international BMI cut off points for different ages [17]. Z-scores were not used because no standard international or Jordanian reference values were available for this age group.

### Procedure

The measurements were conducted at the same time that the questionnaire was circulated and the results were recorded on the questionnaire for each child. The children were given the questionnaires and the consent form to take home to be completed by their mothers and returned, the consent form included a short summary of the study and its objectives. If the mothers were unschooled fathers were asked to help the mothers in completing the form. The measurement of children’s height and weight who had returned a completed consent form and questionnaire was performed by the same researcher at each school. Children’s weight was measured using a Tanita BC543 scale.

Ethical approval for the research was obtained from AlZaytoonah University Research Ethics Committee.

### Statistical analysis

The items were treated as ordinals and the normality of scores on each subscale of each model was assessed by calculating mean, standard deviation and kurtosis values. The score of each subscale for each participant is the mean of the scores of contributing items. Confirmatory factor analysis on the 12-factor model was conducted using AMOS 22 and SPSS 20. Item loading at the designated factors of the suggested 12-factor model were examined and goodness of fit was evaluated by calculating CMIN/DF (minimum discrepancy), GFI (goodness of fit index), TLI (Tucker-Lewis coefficient), CFI (comparative fit index) and RMSEA (Root Mean Square Error of Approximation). Acceptable values for CMIN/DF are 2–5, for RMSEA less than 0.6 and for GFI, CFI and TLI values closer to 1. The cut-off used to determine if items loaded on a factor was 0.4. Finally, correlation between the 12 factors in the suggested model were evaluated using Pearson’s correlation to examine discriminant validity.

The suitability of data for factor analysis was evaluated using the Kaiser-Meyer-Olkin value (KMO) and Bartlett’s Test of Sphericity. Exploratory factor analysis was conducted to evaluate the suitable model for the data after determining that the 12-factor model reported low goodness of fitness indicators. To determine the appropriate number of factors to extract, Parallel Analysis (Eigenvalue Monte Carlo Simulation) was conducted using O’Connor’s SPSS syntax [18], and scree plots were examined; to obtain this a graph was plotted for each eigenvalue in the Y-axis against the factor with which it was associated in the X-axis, then the inflection point was identified and the number of factors are determined as the number of factors present in the curve prior to the inflection point. Pattern matrix was generated using promax method and examined to identify the proper pattern matrix. Communalities represent the multiple correlation between each variable and the factors extracted and it is equal to the sum of squared factor loadings for the variables. A communality below 0.3 indicates that the variable may have little in common with any of the other variables and was dropped from the analysis. The factor correlation matrix was evaluated to determine discriminant validity.

Internal consistency for each subscale was evaluated by calculating Cronbach’s alpha; Cronbach’s alpha above 0.6 were considered acceptable. The final suggested model was evaluated using confirmatory factor analysis. Finally, a generalized mixed logistic regression model with random intercept was performed using the mixed model command (GENLINMIXED) in SPSS.

The regression was modelled by two levels with clustering by school. The model included child weight status (ordinal variable of three levels: normal weight, overweight and obese) as the dependent variable, and the predictors in the model were factors of the final model, maternal BMI, child gender and maternal education level. Model assumptions were checked and included multicollinearity that was evaluated by examining variance inflation factor (VIF) and tolerance values (VIF less than 10 and tolerance greater than 0.2), and proportional odds that was assessed by examining the test of parallel lines (*p* value greater than 0.05). However, the output indicated that the final Hessian Matrix was not positive definite which indicated that there was no variance between different schools and the similarities between children from different schools were the same. Therefore, ordinal logistic regression was performed without the random intercept.

## Results

Completed questionnaires were returned by 970 children with signed consent forms by their parents/guardians. This represented a response rate of 72%. There were 488 boys and 482 girls, with a mean age of 9.1. As seen in Table 2 the children enrolled in the study were distributed almost evenly between the schools. The children had high rates of obesity and overweight and their mothers had high rates of reported obesity and overweight. Although the sample included different maternal education levels ranging from unschooled to PhDs, about half the participants were high school educated (43%) and only 4.1% of them were unschooled. These percentages are comparable to a study that measured the rate of education among Jordanians [19].

**Table 2:** Demographic and anthropometric characteristics

		Number	Percent^a^
Gender	Male	488	50.3
	Female	482	49.7
Age (year)	6	88	9.1
	7	127	13.1
	8	186	19.2
	9	149	15.4
	10	154	15.9
	11	159	16.4
	12	107	11
School	1	208	21.4
	2	188	19.4
	3	172	17.7
	4	210	21.6
	5	192	19.8
Child Weight Classification	Normal	614	63.3
	Overweight	235	24.2
	Obese	121	12.5
Maternal Weight Classification	Normal	266	27.4
	Overweight	334	34.4
	Obese	370	38.1
Maternal Education Level	Unschooled	40	4.1
	Middle school	139	14.3
	High school	424	43.7
	Diploma	188	19.4
	Bachelor Degree	164	16.9
	Master’s degree	10	1.0
	PhD	5	0.5

^a^The sums of many of these % variables do not add up to 100 due to rounding error

When running the CFA of the original model, several items did not load in the designated factor, for example items 13, 18, and 38 did not reach the 0.4 loading cut-off point.

Examining the communalities table of the original 12 factor 49 items showed low communalities in item 13, 24, and 38 (0.13, 0.20 and 0.23 respectively) which are from the *Encourage balance and variety*, item 17 of the *Pressure* factor and item 18 of *Restriction for weight* factor (0.19 and 0.15) and item 42 from the *Teach* factor (0.22). Therefore, all of these items were excluded from the analysis.

The highest correlation was found between *Encourage balance and variety* factor was and *Teaching* factor (*r* = 0.4, *p* < 0.01). After investigating these results, it was clear that the questionnaire in its original form was not fit for this sample and it was decided that EFA should be performed to evaluate the most appropriate questionnaire structure for this sample.

The Kaiser-Meyer-Olkin test result was 0.81 and Bartlett’s Test of Sphericity was significant *χ* ^2^ (1176) = 15803.65, *p* < 0.01 which indicated the suitability of the data for factor analysis. Exploratory factor analysis was rerun after excluding item numbers 13,17, 18, 24, 38 and 42 and scree plots were examined that suggested 11 factors. The 11 factor model was reconfirmed when conducting parallel analysis (Monte Carlo simulation) and examining eigenvalues greater than 1. The 11 factor model included *Modelling, Monitoring, Child control, Food as a reward, Emotional regulation, Involvement, Restriction for health, Restriction for weight control, Environment, Teach and encourage* and *Pressure*. The communalities of the items included in the final 11 factor model were all above 0.3 and the lowest loading was 0.49 (Item 26 in the Teach and encourage subscale: I tell my child that healthy food tastes good) (Table 3).

**Table 3:** Subscale names, item numbers, factor loadings, communalities, and Cronbach’s alpha for the 11-factors model

Subscale name(Item numbers)	Factor loadingsmin-max	Communalitiesmin-max	Cronbach’salpha
Monitoring Item (1, 2, 3, 4)	0.59–0.79	0.38–0.59	0.77
Child control Item (5, 6, 10, 11, 12)	0.60–0.72	0.38–0.55	0.78
Emotion regulation Item (7,8,9)	0.62–0.85	0.43–0.70	0.80
Environment Item (14,16,22,37)	0.63–0.73	0.42–0.58	0.77
Involvement Item (20, 15, 32)	0.52–0.78	0.31–0.61	0.68
Pressure Item (30, 39, 49)	0.61–0.66	0.41–0.45	0.66
Restriction for weight control. Item (27, 29, 33, 34, 35, 41, 45)	0.51–0.65	0.30–0.46	0.79
Food as reward Item (19, 23, 36).	0.59–0.82	0.43–0.68	0.77
Restriction for health Item (21, 28,40, 43).	0.56–0.82	0.49–0.60	0.81
Modelling Item (44, 46, 47, 48)	0.73–0.94	0.57–0.88	0.90
Teach and encourage Item (25, 26, 31)	0.49–0.76	0.37–0.55	0.71

Cronbach’s alpha values were examined and although removing item 46 would improve the *Modelling* subscale from 0.90 to 0.91, it was decided that the benefit of keeping item 46 overweighs the benefit of removing it, because the Cronbach’s alpha value was high even when item 46 was retained and excluding it would only produce 0.01 improvement in Cronbach’s alpha. In addition, increasing the number of items in a subscale improved the model fit (Table 3).

Correlations between factors were examined to determine discriminant validity using Pearson’s correlation (Table 4). The results indicated good discriminant validity (r between 0.02 and .37), the highest correlation was between *Restriction for weight* and *Restriction for health*.

**Table 4:** Inter-factor correlations within the 11-factor model from the confirmatory analysis, *n* = 970

	1	2	3	4	5	6	7	8	9	10	
1 Monitoring	-										
2 Child Control	−0.08^b^	-									
3 Emotional regulation	−0.10^a^	0.19^a^	-								
4 Environment	0.16^a^	−0.03	−0.16^a^	-							
5 Involvement	0.11^a^	0.05	−0.05	0.10^a^	-						
6 Pressure	0.03	0.06	0.25^a^	−0.05	0.04	-					
7 Restriction for weight	0.03	−0.13^a^	−0.01	0.07	0.14^a^	−0.14^a^	-				
8 Food as a reward	−0.06	0.06	0.24^a^	−0.10^a^	0.17^a^	0.23^a^	0.15^a^	-			
9 Restriction for health	0.06	−0.08^b^	−0.08^b^	0.15^a^	0.15^a^	−0.01	0.37^a^	0.02	-		
10 Modelling	0.07^b^	0.02	−0.03	0.07^b^	0.13^a^	0.08^b^	0.11^a^	−0.02	0.11^a^	-	
11 Teach and encourage	0.16^a^	−0.04	−0.13^a^	0.11^a^	0.27^a^	0.02	0.16^a^	0.05	0.19^a^	0.35^a^	-

^a^ Correlation is significant at the 0.01 level (2-tailed)

^b^ Correlation is significant at the 0.05 level (2-tailed)

Confirmatory factor analysis of the suggested 11 factor model with four error covariance yielded acceptable model fit indicators (CMIN/DF = 2.18, GFI = 0.92, CFI = 0.93, TLI = 0.92 and RMSEA = 0.03).

As the regression table (Table 5) shows there were negative correlations between healthy weight status and *Restriction to health, Restriction to weight, Emotional regulation* and maternal BMI, and a positive association with *Monitoring, Modelling, Teach and Encourage, Child control, Involvement* and *Environment*. A Nagelkerke test indicated that 12.5% of variance present in child weight status was explained by this model.

**Table 5:** Ordinal regression of different factors associated with Child weight status (ordinal variable: Normal, overweight and obese), *n* = 970

Parameter	*p*-value	Odds ratio	95% Confidence Interval	
			Lower Bound	Upper Bound
Threshold obese	0.76	1.58	−1.56	2.14
Threshold overweight	0.05	3.14	−0.00	3.7
Monitoring	0.03	0.19	0.20	0.36
Child Control	0.00	0.35	0.19	0.51
Emotional regulation	0.02	−0.18	−0.33	−0.03
Environment	0.01	0.21	0.05	0.38
Involvement	0.02	0.18	0.03	0.35
Pressure	0.54	−0.04	−0.17	0.09
Restriction to weight	0.03	−0.18	−0.35	−0.16
Food as a reward	0.90	0.01	−0.13	0.15
Restriction to health	0.00	−0.28	−0.47	−0.09
Modelling	0.03	0.16	0.01	0.31
Teach and encourage	0.00	0.38	0.17	0.59
Maternal BMI	0.02	−0.03	−0.61	−0.01
PhD	0.18	−1.29	−3.16	0.58
Master’s degree	0.48	0.61	−1.09	2.31
Bachelor Degree	0.79	−1.05	−0.86	0.65
Diploma	0.28	−0.41	−1.14	0.33
Unschooled	0.45	−0.27	−0.97	0.43
Middle school	0.44	−0.3	−1.05	0.46
Not schooled (reference)				
Boys	0.95	−0.01	.-0.28	.0.26
Girls(reference)				

## Discussion

This study developed and evaluated an Arabic form of the Comprehensive Feeding Practice Questionnaire, and evaluated the association between each factor and child weight. This study evaluated maternal feeding practices because mothers are usually the parent responsible for the children’s feeding; this is common globally but even more so in Jordan and throughout the Arab world. Therefore, several studies have focused on maternal feeding practices and did not evaluate the paternal role [20–22].

The original 12 factor model showed low fit indication for our sample. The exploratory factor analysis yielded an 11 factor model constructed from 43 items. Several studies conducted in different parts of the world have proposed different models of the CFPQ. For example, a study that used the Portuguese version of CFPQ among Brazilian parents suggested a six factor model that included 42 items [23], a study that validated the questionnaire among Norwegian parents suggested a 10 factor model constructed from 42 items [24] and one conducted in New Zealand proposed a five factor model that was constructed from 32 items, the five suggested scales were: Healthy Eating Guidance, Monitoring, Parent Pressure, Restriction and Child Control [25]. The Iranian validated version was composed of 12 factor model constructed from 46 questions [26] and Malaysian version contained 12-factor model with 39 items [27].

The differences between various validated versions of the CFPQ in different countries could be attributed to methodological differences between the different studies. For example, the Portuguese study was performed on preschool children, while our study was performed on older school aged children. In addition, cultural and social differences could influence the final validated versions of the questionnaire.

As expected the results of the EFA resembled the original 12 factor model and most of the items loaded in their designated factor; 10 factors of our 11 factor model were similar to the original model, these factors *were Modelling, Monitoring, Child control, Restriction to weight, Emotional regulation, Involvement, Restriction for health, Restriction for weight control*, and *Environment* [8], while the last one was composed from items from the original *Teach* subscale and *Encourage balance and variety* subscale. Items from these two scales were included as a single factor in previous studies [23, 25]. These two factors were significantly correlated (*r* = 0.3) in the original Musher-Eizenman and Holub model [8] and in Melbye’s model (*r* = 0.5) [24], perhaps because parents who use positive practice habits usually use them in combination with other practices [28]. This also may explain several significant correlations between positive feeding practices including between *Teach and encourage* and *Monitoring*, *Teach and Encourage* and *Environment, Teach and encourage* and *Involvement, Teach and encourage* and *Modelling, Environment* and *Monitoring* and *Involvement* and *Modelling*. Several significant negative associations were found between negative feeding practices and positive ones including between *Emotional regulation* and *Monitoring*, and *Emotional regulation* and *Environment.*

The highest correlation in our model was between *Restriction to Health* and *Restriction to Weight* (*r* = 0.37) which indicated good discriminant validity. The strong association between these two factors has also been reported in previous work [23], perhaps because the parents are not always clear about their motivation for restriction [24].

The lowest Cronbach’s alpha reported in this study was 0.66 in the Pressure subscale which was higher than some low Cronbach’s alphas reported in Musher-Eizenman and Holub’s model [8] and by Musher-Eizenman et al. [29]. Although the recommended acceptable Cronbach’s alpha value is usually 0.7, the value of Cronbach’s alpha depends on the number of items in the factor (scale). When the number of items in the factor is less than 10 as in this study, acceptable Cronbach’s alpha values can be less than 0.7 [30].

### Association between feeding practice and children’s weight

The regression showed that feeding practices were associated with child obesity status. *Restriction for weight control* and *Restriction for health* were associated with higher child weight status and has been reported in previous studies [31–33]. Several hypotheses have forwarded been to explain this association. One hypothesis suggests that denying children specific types of food makes them more desirable and will lead children to overconsume those foods when possible which may eventually lead to an increase in their BMI [34]. This was supported by a study that found relationship between restrictive feeding practices and eating in the absence of hunger [7]. A further hypothesis suggests that parents will tend to restrict food intake for obese/overweight children more than normal weight children. This is supported by work that found that restriction increases after weight gain, not before it [20]*. Emotional regulation* was also associated with increased child weight status. Children of mothers who use *Emotional regulation* consume more chocolate and cookies to elevate distress even in the absence of hunger than other children [35] which may explain our finding.

All the positive feeding practices were positively associated with healthy weight status. This is consistent with other work that found negative associations between *Monitoring* and BMI [5], although another study has reported an opposite finding [6] and indicated a positive association between child BMI and *Monitoring*. Such conflicting findings reported between *Monitoring* and other parental feeding practices could be attributed to specific characteristics of the children including the way they react to *Monitoring* and other feeding practices [4]. Furthermore, cultural diversity may influence the way children react to different feeding practices. Therefore, the impact of different feeding practices on children should be evaluated in different countries. As in this study, other work reported that *Involvement* was associated with lower child BMI [36]. *Child control* of their food was also associated with healthy weight status which supports recommendations to use *Child control* to prevent and treat child obesity [37, 38].

Although *Pressure to eat* and *Food as a reward* were not significantly associated with child weight status in this study, other studies have reported an increase in *Pressure to eat* practice in children with lower BMI [39], and a negative association between *food as a reward* and increase in child BMI [21].

### Limitations

There were several limitations. First, cognitive interviews were not performed, which could have affected the way participants interpreted the questions. However, the questionnaire was translated into Arabic and back translated by a different person. The back translated version was compared with the original English questionnaire and the accuracy of the back translated version indicates that the Arabic version was clear and understandable for the back translator. In addition, the high internal consistency indicates that the questions were clear for the respondents.

Second, unschooled mothers had to rely on fathers when completing the questionnaires. However, the percentage of the unschooled mothers was only 4.1% of the total sample and no significant differences were found in the internal consistency between the unschooled mothers and the rest of the sample.

Finally, as with other similar studies there is always the issue of social desirability bias as some parents may be under the pressure to report a higher rate of healthy feeding practices [40].

## Conclusion

The Arabic version of the CFPQ provides an adequate tool to investigate childhood overweight and obesity in the Middle East region, which can be utilized in investigating and developing interventions to tackle the childhood overweight and obesity in the area. This study indicated overweight and obesity in children were associated with negative maternal feeding practices.

## Additional files

Additional file 1: Arabic version of the CFPQ. (DOCX 24.6 kb)
Additional file 2: Original English version of the CFPQ. (DOCX 24.2 kb)
