# Supplementary material for: Validation of the comprehensive feeding practice questionnaire among school aged children in Jordan: a factor analysis study
Source: Int J Behav Nutr Phys Act. 2017 Feb 20;14:23. doi: 10.1186/s12966-017-0478-y (PMC5319026; doi:10.1186/s12966-017-0478-y)
Supplement: Additional file 1: — Arabic version of the CFPQ. (DOCX 24.6 kb) [file 12966_2017_478_MOESM1_ESM.docx]

**Additional file 1**

**Arabic version of the CFPQ**

عزيزتي الام نرجو من حضرتك المشاركة بهذا الاستبيان القصير بهدف التعرف على الانماط والسلوك الغذائية لأطفالنا, نتائج هذا الاستبيان ستكون مفيدة جدا للصحة العامة للمجتمع

وزن الام_______________________________

طول الام_______________________________

مستوى الام التعليمي________________________

عمر الطفل_____________________________

وزن الطفل_______________________________

طول الطفل______________________________

1-لاي مدى تراقبي كميات الحلويات التي يستهلكها الطفل (كعك, بوظة, شوكولاتة, سكاكر)؟

دائما ☐ غالبا ☐احيانا ☐ نادرا ☐ ابدا ☐

2-لاي مدى تراقبي كميات الوجبات الخفيفة (سناك) التي يستهلكها الطفل (شيبس، رقائق الدرة-بفك-)؟

دائما ☐ غالبا ☐احيانا ☐ نادرا ☐ ابدا ☐

3- لأي مدى تراقبي كميات الاطعمة الدسمة (عالية الدهون) التي يستهلكها الطفل؟

دائما ☐ غالبا ☐احيانا ☐ نادرا ☐ ابدا ☐

4- لأي مدى تراقبي كميات المشروبات الغنية بالسكر اللتي يستهلكها الطفل(بيبسي عصائر البودرة مثل التانج, سيفن)؟

دائما ☐ غالبا ☐احيانا ☐ نادرا ☐ ابدا ☐

5-هل تدعي الطفل يأكل اي شيء يريده؟

دائما ☐ غالبا ☐احيانا ☐ نادرا ☐ ابدا ☐

6-في وجبة الغذاء هل تدعي الطفل يختار اي صنف يريد من انواع الاصناف المقدمة؟

دائما ☐ غالبا ☐احيانا ☐ نادرا ☐ ابدا ☐

7-عندما يكون الطفل منزعج هل اعطاءه شيء للأكل هو اول ما تفعليه؟

دائما ☐ غالبا ☐احيانا ☐ نادرا ☐ ابدا ☐

8-هل تعطي الطفل طعاما إذا كان الطفل يشعر بالملل حتى لو لم يكن الطفل جائع؟

دائما ☐ غالبا ☐احيانا ☐ نادرا ☐ ابدا ☐

9- هل تعطي الطفل طعاما إذا كان الطفل مستاء او منزعج حتى لو لم يكن الطفل جائع؟

دائما ☐ غالبا ☐احيانا ☐ نادرا ☐ ابدا ☐

10-إذا لم يعجب الطفل الطعام المقدم هل تحضرين له نوع آخر من الاطعمة؟

دائما ☐ غالبا ☐احيانا ☐ نادرا ☐ ابدا ☐

11-هل تسمح للطفل بأكل الوجبات الخفيفة متى شاء؟

دائما ☐ غالبا ☐احيانا ☐ نادرا ☐ ابدا ☐

12-هل تسمحين للطفل بترك مائدة الطعام اذا احس بالشبع حتى لو لم تنتهي العائلة من الاكل؟

دائما ☐ غالبا ☐احيانا ☐ نادرا ☐ ابدا ☐

13-هل تشجعين الطفل لأكل الطعام الصحي قبل الطعام الغير صحي؟

دائما ☐ غالبا ☐احيانا ☐ نادرا ☐ ابدا ☐

14-هل معظم الطعام الذي تحتفظين به بالبيت هو طعام صحي

أوافق☐ أوافق نوعا ما ☐ ليس لدي جواب (محايد) ☐ ارفض نوعا ما ☐ ارفض ☐

15-انا أخذ رأي الطفل في اختيار وجبات العائلة (مثال: ماذا تحب ان اطبخ اليوم)؟

أوافق☐ أوافق نوعا ما ☐ ليس لدي جواب (محايد) ☐ ارفض نوعا ما ☐ ارفض ☐

16-احتفظ بالكثير من اطعمة الوجبات الخفيفة في البيت مثل ( الشيبس , رقائق الدرة-بفك-)؟

ارفض☐ ارفض نوعا ما ☐ ليس لدي جواب (محايد) ☐ أوافق نوعا ما ☐ أوافق ☐

17-يجب على طفلي ان يأكل دائما كل ما في صحنه من طعام

أوافق☐ أوافق نوعا ما ☐ ليس لدي جواب (محايد) ☐ ارفض نوعا ما ☐ ارفض ☐

18-يجب ان اكون متأكدة ان طفلي لا يأكل كميات كبيرة من الاطعمة الدسمة (عالية الدهون)

أوافق☐ أوافق نوعا ما ☐ ليس لدي جواب (محايد) ☐ ارفض نوعا ما ☐ ارفض ☐

19-انا أقدم لطفلي اكله المفضل كمكافئه على تصرفاته الجيدة

أوافق☐ أوافق نوعا ما ☐ ليس لدي جواب (محايد) ☐ ارفض نوعا ما ☐ ارفض ☐

20-اسمح لطفي بالمشاركة في تحضير الطعام

أوافق☐ أوافق نوعا ما ☐ ليس لدي جواب (محايد) ☐ ارفض نوعا ما ☐ ارفض ☐

21-إذا لم اقم بتوجيه طفلي او انظم ما يأكله سيأكل الكثير من اكله المفضل

أوافق☐ أوافق نوعا ما ☐ ليس لدي جواب (محايد) ☐ ارفض نوعا ما ☐ ارفض ☐

22-اطعمة صحية متنوعة تقدم لطفلي بكل وجبة في المنزل

أوافق☐ أوافق نوعا ما ☐ ليس لدي جواب (محايد) ☐ ارفض نوعا ما ☐ ارفض ☐

23-انا اقدم لطفلي الحلويات (كعك، بوظة, شوكولاتة, سكاكر) كمكافئه على تصرفاته الجيدة

أوافق☐ أوافق نوعا ما ☐ ليس لدي جواب (محايد) ☐ ارفض نوعا ما ☐ ارفض ☐

24-انا أشجع طفلي ليجرب اطعمة جديدة

أوافق☐ أوافق نوعا ما ☐ ليس لدي جواب (محايد) ☐ ارفض نوعا ما ☐ ارفض ☐

25-انا اتناقش مع طفلي حول اهمية اكل الانواع الصحية من الطعام

أوافق☐ أوافق نوعا ما ☐ ليس لدي جواب (محايد) ☐ ارفض نوعا ما ☐ ارفض ☐

26-انا أخبر طفلي ان الطعام الصحي لذيذ

أوافق☐ أوافق نوعا ما ☐ ليس لدي جواب (محايد) ☐ ارفض نوعا ما ☐ ارفض ☐

27-انا أشجع طفلي على تقليل اكله لكي لا يصبح سمينا

أوافق☐ أوافق نوعا ما ☐ ليس لدي جواب (محايد) ☐ ارفض نوعا ما ☐ ارفض ☐

28-اذا لم انظم ما يأكله طفلي سيأكل اكثر من اللازم من الوجبات السريعة (هامبرجر, بيتزا, شاورما)

أوافق☐ أوافق نوعا ما ☐ ليس لدي جواب (محايد) ☐ ارفض نوعا ما ☐ ارفض ☐

29-أقدم لطفلي حصة صغيرة من الطعام في الوجبات لأتحكم بوزنه

أوافق☐ أوافق نوعا ما ☐ ليس لدي جواب (محايد) ☐ ارفض نوعا ما ☐ ارفض ☐

30-حتى إذا طفلي قال انه ليس جائعا سأحاول ان اطعمه

أوافق☐ أوافق نوعا ما ☐ ليس لدي جواب (محايد) ☐ ارفض نوعا ما ☐ ارفض ☐

31-اناقش مع طفلي القيم الغذائية للطعام

أوافق☐ أوافق نوعا ما ☐ ليس لدي جواب (محايد) ☐ ارفض نوعا ما ☐ ارفض ☐

32-انا اشجع طفلي ليشارك في شراء المواد الغذائية

أوافق☐ أوافق نوعا ما ☐ ليس لدي جواب (محايد) ☐ ارفض نوعا ما ☐ ارفض ☐

33-إذا طفلي اكل أكثر من العادة في احدى الوجبات سأقيد (احدد) له كمية الطعام في الوجبة التي تليها

أوافق☐ أوافق نوعا ما ☐ ليس لدي جواب (محايد) ☐ ارفض نوعا ما ☐ ارفض ☐

34-انا اقيد (احدد) من انواع الطعام التي ممكن ان تجعل طفلي سمينا

أوافق☐ أوافق نوعا ما ☐ ليس لدي جواب (محايد) ☐ ارفض نوعا ما ☐ ارفض ☐

35-بعض انواع من الاكل لا يجب على طفلي اكلها لأنها ستجعله سمين

أوافق☐ أوافق نوعا ما ☐ ليس لدي جواب (محايد) ☐ ارفض نوعا ما ☐ ارفض ☐

36-انا احرم طفلي من الحلويات والتحلية ( التحلاية) كرة فعل على التصرفات السيئة

أوافق☐ أوافق نوعا ما ☐ ليس لدي جواب (محايد) ☐ ارفض نوعا ما ☐ ارفض ☐

37-انا احتفظ بالكثير من الحلويات (كعك, بوظة, شوكولاتة, سكاكر) في المنزل

ارفض☐ ارفض نوعا ما ☐ ليس لدي جواب (محايد) ☐ أوافق نوعا ما ☐ أوافق ☐

38-انا أشجع طفلي ان يأكل اكلا متنوعا

أوافق☐ أوافق نوعا ما ☐ ليس لدي جواب (محايد) ☐ ارفض نوعا ما ☐ ارفض ☐

39-اذا طفلي اكل حصة صغيرة من الطعام سأجاول ان اجعله يأكل المزيد

أوافق☐ أوافق نوعا ما ☐ ليس لدي جواب (محايد) ☐ ارفض نوعا ما ☐ ارفض ☐

40-يجب ان احرص على ان طفلي لا يأكل أكثر من اللازم من اكله المفضل

أوافق☐ أوافق نوعا ما ☐ ليس لدي جواب (محايد) ☐ ارفض نوعا ما ☐ ارفض ☐

41-لا اسمح لطفلي ان يأكل بين الوجبات لأني لا اريد ان يصبح سمينا

أوافق☐ أوافق نوعا ما ☐ ليس لدي جواب (محايد) ☐ ارفض نوعا ما ☐ ارفض ☐

42-انا اخبر طفلي ماذا يجب عليه ان يأكل وماذا يجب عليه ان لا يأكل بدون تفسير الاسباب

ارفض☐ ارفض نوعا ما ☐ ليس لدي جواب (محايد) ☐ أوافق نوعا ما ☐ أوافق ☐

43-يجب ان أتأكد بالا يأكل طفلي اكثر من اللازم من الحلويات (سكاكر, بوظة , كيك ).

أوافق☐ أوافق نوعا ما ☐ ليس لدي جواب (محايد) ☐ ارفض نوعا ما ☐ ارفض ☐

44- انا اكل اكلا صحيا لأكون قدوة لابني في الاكل الصحي.

أوافق☐ أوافق نوعا ما ☐ ليس لدي جواب (محايد) ☐ ارفض نوعا ما ☐ ارفض ☐

45- غالبا ما اقوم بعمل حمية لطفلي للمحافظة على وزنه\ها

أوافق☐ أوافق نوعا ما ☐ ليس لدي جواب (محايد) ☐ ارفض نوعا ما ☐ ارفض ☐

46- احاول اكل اطعمة صحية امام طفلي حتى لو لم تكن مفضلة لدي.

أوافق☐ أوافق نوعا ما ☐ ليس لدي جواب (محايد) ☐ ارفض نوعا ما ☐ ارفض ☐

47- احاول ان اظهر حماسا في اكل الاطعمة الصحية

أوافق☐ أوافق نوعا ما ☐ ليس لدي جواب (محايد) ☐ ارفض نوعا ما ☐ ارفض ☐

48- اظهر لطفلي مدى استمتاعي بأكل الاطعمة الصحية

أوافق☐ أوافق نوعا ما ☐ ليس لدي جواب (محايد) ☐ ارفض نوعا ما ☐ ارفض ☐

49- عندما يقول طفلي انه انتهى من الاكل احاول اقناعه بتناول المزيد من الطعام (لقمة اضافية , لقمتين ..... الخ).

أوافق☐ أوافق نوعا ما ☐ ليس لدي جواب (محايد) ☐ ارفض نوعا ما ☐ ارفض ☐
