# Supplementary material for: Validation of the comprehensive feeding practice questionnaire among school aged children in Jordan: a factor analysis study
Source: Int J Behav Nutr Phys Act. 2017 Feb 20;14:23. doi: 10.1186/s12966-017-0478-y (PMC5319026; doi:10.1186/s12966-017-0478-y)
Supplement: Additional file 2: — Original English version of the CFPQ. (DOCX 24.2 kb) [file 12966_2017_478_MOESM2_ESM.docx]

**Original English version of the CFPQ**

| Parents take many different approaches to feeding their children and may have different concerns about feeding depending on their child. **Please answer the following questions as honestly as possible with this child in mind.** | Never | Rarely | Sometimes | Mostly | Always |
| --- | --- | --- | --- | --- | --- |
| 1. How much do you keep track of the sweets (candy, ice cream, cake, pies, pastries) that your child eats? | 1 | 2 | 3 | 4 | 5 |
| 1. How much do you keep track of the snack food (potato chips, Doritos, cheese puffs) that your child eats? | 1 | 2 | 3 | 4 | 5 |
| 1. How much do you keep track of the high-fat foods that your child eats? | 1 | 2 | 3 | 4 | 5 |
| 1. How much do you keep track of the sugary drinks (soda/pop, Kool-Aid) this child drinks? | 1 | 2 | 3 | 4 | 5 |
| 1. Do you let your child eat whatever s/he wants? | 1 | 2 | 3 | 4 | 5 |
| 1. At dinner, do you let this child choose the foods s/he wants from what is served? | 1 | 2 | 3 | 4 | 5 |
| 1. When this child gets fussy, is giving him/her something to eat or drink the *first* thing you do? | 1 | 2 | 3 | 4 | 5 |
| 1. Do you give this child something to eat or drink if s/he is bored even if you think s/he is not hungry? | 1 | 2 | 3 | 4 | 5 |
| 1. Do you give this child something to eat or drink if s/he is upset even if you think s/he is not hungry? | 1 | 2 | 3 | 4 | 5 |
| 1. If this child does not like what is being served, do you make something else? | 1 | 2 | 3 | 4 | 5 |
| 1. Do you allow this child to eat snacks whenever s/he wants? | 1 | 2 | 3 | 4 | 5 |
| 1. Do you allow this child to leave the table when s/he is full, even if your family is not done eating? | 1 | 2 | 3 | 4 | 5 |
| 1. Do you encourage this child to eat healthy foods before unhealthy ones? | 1 | 2 | 3 | 4 | 5 |

|  | Disagree | Slightly disagree | Neutral | Slightly agree | Agree |
| --- | --- | --- | --- | --- | --- |
| 1. Most of the food I keep in the house is healthy. | 1 | 2 | 3 | 4 | 5 |
| 1. I involve my child in planning family meals. | 1 | 2 | 3 | 4 | 5 |
| 1. I keep a lot of snack food (potato chips, Doritos, cheese puffs) in my house. | 1 | 2 | 3 | 4 | 5 |
| 1. My child should always eat all of the food on his/her plate. | 1 | 2 | 3 | 4 | 5 |
| 1. I have to be sure that my child does not eat too many high-fat foods. | 1 | 2 | 3 | 4 | 5 |
| 1. I offer my child his/her favorite foods in exchange for good behavior. | 1 | 2 | 3 | 4 | 5 |
| 1. I allow my child to help prepare family meals. | 1 | 2 | 3 | 4 | 5 |
| 1. If I did not guide or regulate my child’s eating, s/he would eat too much of his/her favorite foods. | 1 | 2 | 3 | 4 | 5 |
| 1. A variety of healthy foods are available to my child at each meal served at home. | 1 | 2 | 3 | 4 | 5 |
| 1. I offer sweets (candy, ice cream, cake, pastries) to my child as a reward for good behavior. | 1 | 2 | 3 | 4 | 5 |
| 1. I encourage my child to try new foods. | 1 | 2 | 3 | 4 | 5 |
| 1. I discuss with my child why it’s important to eat healthy foods. | 1 | 2 | 3 | 4 | 5 |
| 1. I tell my child that healthy food tastes good. | 1 | 2 | 3 | 4 | 5 |
| 1. I encourage my child to eat less so he/she won’t get fat. | 1 | 2 | 3 | 4 | 5 |
| 1. If I did not guide or regulate my child’s eating, s/he would eat too many junk foods. | 1 | 2 | 3 | 4 | 5 |
| 1. I give my child small helpings at meals to control his/her weight. | 1 | 2 | 3 | 4 | 5 |
| 1. If my child says, “I’m not hungry,” I try to get him/her to eat anyway. | 1 | 2 | 3 | 4 | 5 |
| 1. I discuss with my child the nutritional value of foods. | 1 | 2 | 3 | 4 | 5 |
| 1. I encourage my child to participate in grocery shopping. | 1 | 2 | 3 | 4 | 5 |
| 1. If my child eats more than usual at one meal, I try to restrict his/her eating at the next meal. | 1 | 2 | 3 | 4 | 5 |
| 1. I restrict the food my child eats that might make him/her fat. | 1 | 2 | 3 | 4 | 5 |
| 1. There are certain foods my child shouldn’t eat because they will make him/her fat. | 1 | 2 | 3 | 4 | 5 |
| 1. I withhold sweets/dessert from my child in response to bad behavior. | 1 | 2 | 3 | 4 | 5 |
| 1. I keep a lot of sweets (candy, ice cream, cake, pies, pastries) in my house. | 1 | 2 | 3 | 4 | 5 |
| 1. I encourage my child to eat a variety of foods. | 1 | 2 | 3 | 4 | 5 |
| 1. If my child eats only a small helping, I try to get him/her to eat more. | 1 | 2 | 3 | 4 | 5 |
| 1. I have to be sure that my child does not eat too much of his/her favorite foods. | 1 | 2 | 3 | 4 | 5 |
| 1. I don’t allow my child to eat between meals because I don’t want him/her to get fat. | 1 | 2 | 3 | 4 | 5 |
| 1. I tell my child what to eat and what not to eat without explanation. | 1 | 2 | 3 | 4 | 5 |
| 1. I have to be sure that my child does not eat too many sweets (candy, ice cream, cake, or pastries). | 1 | 2 | 3 | 4 | 5 |
| 1. I model healthy eating for my child by eating healthy foods myself. | 1 | 2 | 3 | 4 | 5 |
| 1. I often put my child on a diet to control his/her weight. | 1 | 2 | 3 | 4 | 5 |
| 1. I try to eat healthy foods in front of my child, even if they are not my favorite. | 1 | 2 | 3 | 4 | 5 |
| 1. I try to show enthusiasm about eating healthy foods. | 1 | 2 | 3 | 4 | 5 |
| 1. I show my child how much I enjoy eating healthy foods. | 1 | 2 | 3 | 4 | 5 |
| 1. When he/she says he/she is finished eating, I try to get my child to eat one more (two more, etc.) bites of food. | 1 | 2 | 3 | 4 | 5 |
